# Morphometric evaluation of bone regeneration in segmental mandibular bone defects filled with bovine bone xenografts in a split-mouth rabbit model

**DOI:** 10.1186/s40729-019-0187-1

**Published:** 2019-09-10

**Authors:** Mariana Quirino Silveira Soares, Jeroen Van Dessel, Reinhilde Jacobs, Renato Yassutaka Faria Yaedú, Eduardo Sant’Ana, Danilo da Silva Corrêa, Maria Fernanda Conceição Madeira, Marco Antônio Húngaro Duarte, Izabel Regina Fischer Rubira-Bullen

**Affiliations:** 1 Department of Surgery, Stomatology, Pathology and Radiology, Bauru School of Dentistry, University of São Paulo, Bauru, 17012-901 Brazil; 2 OMFS-IMPATH research group, Department of Imaging and Pathology, Faculty of Medicine, KU Leuven and Oral and Maxillofacial Surgery, University Hospitals Leuven, Leuven, Belgium; 3 Department of Dental Medicine, Karolinska Institutet, Stockholm, Sweden; 4 Department of Dentistry, Endodontics and Dental Materials, Bauru School of Dentistry, University of São Paulo, Bauru, Brazil

**Keywords:** Microcomputed tomography, Alveolar bone grafting, Bone morphometry, Deproteinized bovine bone mineral

## Abstract

**Background:**

Bovine bone grafts have been widely used in dentistry for guided tissue regeneration and can support new bone formation in direct contact with the graft. The aim of this study was to compare the morphometric and bone density changes after using two different bovine bone graft blocks in segmental osseous defects in the mandible of rabbits following different postoperative periods.

**Material and methods:**

Critical size segmental defects were surgically created bilaterally in the jaw of 18 rabbits. The defects were filled with either deproteinized bovine bone mineral with 10% collagen (DBBM-C; BioOss Collagen®), lyophilized bovine medullary bone (LBMB; Orthogen®), or left untreated according to a split-mouth design. Animals were sacrificed after 3 or 6 months of healing. The hemimandibles were scanned ex vivo using a high-resolution (19 μm) microcomputed tomography. Morphometric and bone density parameters were calculated in the region of the defect using CT-Analyser (Bruker). Initial graft blocks were used as baseline.

**Results:**

DBBM-C presented a denser microarchitecture, in comparison to LBMB at baseline. DBBM-C and LBMB grafted regions showed a similar progressive remodeling, with a significant decrease in structure complexity and maintenance of bone volume fraction during the postoperative follow-up periods. Both graft materials showed an enhanced bone replacement and more complex structure compared to untreated defects. The apparent fusion between the graft and host bone was observed only in the defects filled with LBMB.

**Conclusion:**

LBMB grafts showed a similar behavior as DBBM-C regarding structural remodeling. In LBMB samples, apparent integration between the host bone and the graft was present.

## Introduction

Bone grafting is a surgical procedure that replaces missing bone with natural bone transplants (autografts, allografts, xenografts) or synthetic materials (alloplasts) [1, 2]. Autografts are harvested from a donor site in the same individual and transplanted to another site. Autografts induce bone formation with no host response and have been historically thought to be the “gold standard” for bone grafting [3, 4]. However, there are concerns about donor site morbidity, limited bone volume, and the replacement rate can be unpredictable [4].

Xenografts are obtained from animal bone and are widely used for maxillofacial applications [1, 2, 5, 6]. The xenograft most commonly used in bone regeneration procedures is the deproteinized bovine bone mineral (DBBM). This bone graft is submitted to heat (up to 300 °C) and chemical treatments. After this process, the inorganic phase of bovine bone mainly consists of hydroxyapatite that provides the porous structure [5, 7]. In order to form a scaffold block, DBBM particles can be cohesively bound with 10% biodegradable collagen matrix of porcine origin (DBBM-C) [8, 9]. Several research papers have shown the efficacy of DBDM-C in different surgical situations [8–13].

The lyophilized bovine medullary bone (LBMB) graft is another bone block of bovine origin that differs from DBBM in the way of processing. LBMB is first chemically treated, freeze-dried, and afterwards sterilized with gamma radiation. Hereby preserving the original trabecular bone structure and organic composition consisting of type 1 collagen and hydroxyapatite [14, 15]. Previous studies have shown that LBMB is biocompatible, osteoconductive, and has a low resorption rate [16, 17]. Additionally, no toxicity or immunogenic reactions have been reported [14, 16, 17]. Clinical reports have indicated its potential for alveolar bone defects rehabilitation [18] and sinus floor augmentation [17]. However, limited evidence is available regarding the efficacy and resorption rate of LBMB in comparison to other bone graft materials [16, 19].

The main purpose of bone graft materials is to fill the osseous defect and provide a three-dimensional spatial structure harboring cells and tissues that will colonize the graft during healing process [1, 20, 21]. In this way, the geometry and architecture of these scaffolds are critical factors that may affect bone regeneration [22]. Bone graft morphometric parameters, such as pore size, pore shape, interconnectivity, and bone surface volume density, can affect cell adhesion, proliferation, distribution, and graft resorption, thereby directly influencing the bone regeneration [1, 23]. Additionally, bone microarchitecture at the grafte site may also play a role in the following functional rehabilitation [24]. Therefore, the aim of the present study was to investigate by means of the microcomputed tomography (micro-CT) the morphometric characteristics and bone mineral density of the two bovine bone blocks (DBBM-C and LBMB) who underwent different processing methods. Secondly, the bone replacement, resorption rate, and mineral density of both xenografts were compared with untreated experimental mandibular bone defects following 3- or 6-months postoperative period.

## Methods

### Animals and surgical procedure

This study was approved by the Bauru School of Dentistry Animal Ethics Committee (03/2015). Eighteen male adult rabbits weighing 3.5–4.0 kg were included in this study and housed in individual cages. Food and water were ad libitum. The rabbits were randomly divided based on time of sacrifice into 3 (T1) and 6 months (T2) group (nine animals each).

**Fig. 1:**
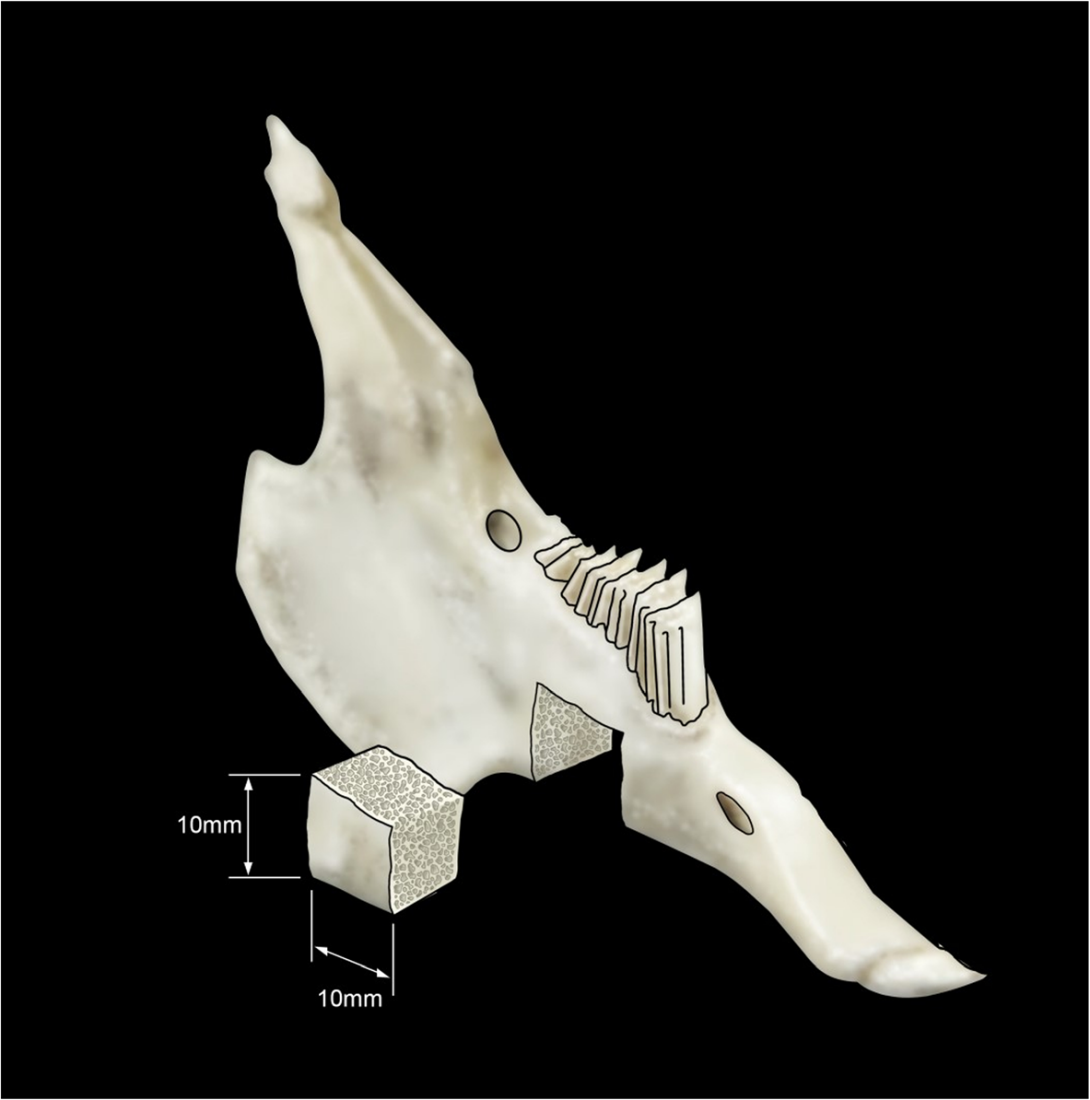
Schematic drawing showing the segmental bone defect in the rabbit’s jaw. The defects were made bilaterally. The segmental mandibular bone defects were made bilaterally and filed with either deproteinized bovine bone mineral with collagen (DBBM-C; Bio-oss^®^) or lyophilized bovine medullary bone (LBMB; Orthogen^®)^

The animals were weighted and anesthetized with a solution of Xylazine (Anasedan^®^, Ceva, Paulínia, Brazil) 10 mg/kg and Ketamine (Dopalen^®^, Vetbrands, Vinhedo, Brazil) 50 mg/kg intramuscularly. The surgical site was shaved and cleaned with povidone-iodine. Local anesthesia was performed with Articaine hydrochloride with adrenaline acid tartrate (Articaíne 4% 1:100000^®^, Nova DFL, Curicica, Brazil). A central incision following the basal cortical of the mandible was performed. Following this procedure, the tissue was dissected; the periosteum excised and detached in the buccal and lingual surfaces; and the bone exposed. Using a conic drill mounted in low-speed handpiece with extensive irrigation with saline solution, a segmental defect (1 × 1 cm) which did not interrupt the mandibular continuity and involved both the lingual and buccal cortical bone was created in mandibular body, immediately anterior to the antegonial notch (Fig. 1). In 12 rabbits, a random split-mouth design was applied and defects were filled in one side with DBBM-C blocks (Bio-Oss Collagen^®^; Geistlich, Wolhusen, Switzerland) and in the other side with LBMB blocks (Orthogen^®^; Genius; Baumer, Mogi Mirim, Brazil). In six animals, the bilateral defects were left untreated. Holes were drilled at an approximate distance of 3 mm from the bone defect and a 0.3 mm stainless steel wire (Aciflex^®^, Ethicon, Johnson & Johnson, New Brunswick, USA) was placed to stabilize the graft to the defect. Following this procedure, the region of the defect was covered with a resorbable collagenous membrane (Osseoguard^®^, Biomet 3i, Palm Beach Gardens, USA). The periosteum and the soft tissues were replaced and sutured with a resorbable wire (Vycril^®^, Ethicon, Jhonson & Jhonson, New Brunswick, USA).

Immediately after surgery, rabbits were given antibiotic (0.6 mg of Shotapen® L.A., Virbac) and 1.2 mg of Ketoprofen (Ketofen® 1%, Merial Saúde Animal, Paulínia, Brazil) intramusculary. The Ketoprofen was administered consecutively for 3 days and the antibiotic dose was repeated after 3 days from surgery. The wounds were inspected daily for clinical signs of complications.

At sacrifice, the animals were first sedated with an intramuscular injection of Xylazine (10 mg/kg) and ketamine (50 mg/kg). Euthanasia was performed with an injection of three times higher dose of the sedation solution. The bone defects and surrounding tissue were harvested.

### Micro-CT

Samples were wrapped with a damp filter paper to avoid dehydration during scanning and placed in a cylindrical sample holder. Scans were acquired using SkyScan1174 machine (Bruker, Kontich, Belgium) with the following scanning parameters: 19 μm^3^ voxel size, 50 kVp, 800 μA, frame averaging of 6, 180° rotations with an angular step of 0.8°. A 0.5 mm aluminum filter was used to reduce artifacts. With the same settings, a water phantom was scanned to allow bone mineral density (BMD) calibration. Four LBMB and DBBM-C blocks were scanned to characterize de initial structure of the blocks (T0). The micro-CT scans were reconstructed using the N-Recon software (Bruker, Kontich, Belgium) and the images were manually aligned using Dataviewer (Bruker, Kontich, Belgium). A quantitative morphometric analysis was performed in CT-Analyzer (Bruker, Kontich, Belgium). A volume of interest (VOI) with the size of the defect was selected and the bone inside the VOI was segmented using an automatic adaptive threshold. Morphometric indices were calculated and grouped according to terms clinically used for bone graft evaluation [25]: (1) *Bone replacement*: bone volume fraction (BV/TV in %), bone surface density (BS/TV in mm^3^/mm^2^), and trabecular thickness (Tb.Th in mm); (2) *Structural remodeling*: trabecular number (Tb.N in 1/mm), trabecular separation (Tb.Sp in mm), connectivity density (Conn.Dn in 1/mm^3^), total porosity percentage (Po[tot] in %), trabecular pattern factor (Tb.Pf in1/mm), structure model index (SMI); and (3) *Bone density* (BMD in mg HA/cm^3^).

### Statistical methods

Descriptive analysis expressed data as mean and standard deviation. The Shapiro-Wilk test was carried out to evaluate data normality. To fully consider the effects within the split-mouth design, two separate general linear models (GLM) were estimated to examine the morphometric differences of bone graft type (DBBM-C; LBMB) and time (T0, T1, T2); and group (untreated, DBBM-C, LBMB) and time (T1, T2) for bone replacement, structural remodeling, and mineral density parameters. Post-hoc Bonferroni corrected *t* tests were used to explore significant interaction effects (α = 0.05). Statistical analysis was performed in SPSS (IBM, New York, USA).

## Results

### General observations

All animals recovered well from surgery and remained in good health during the rest of the experiment. The surgical procedures and follow-up showed no complications regarding the protocol. At T1 and T2, radiographic signs of inflammatory lesions were observed in one sample of LBMB and in one sample of DBBM-C.

### Graft block morphometry

**Fig. 2:**
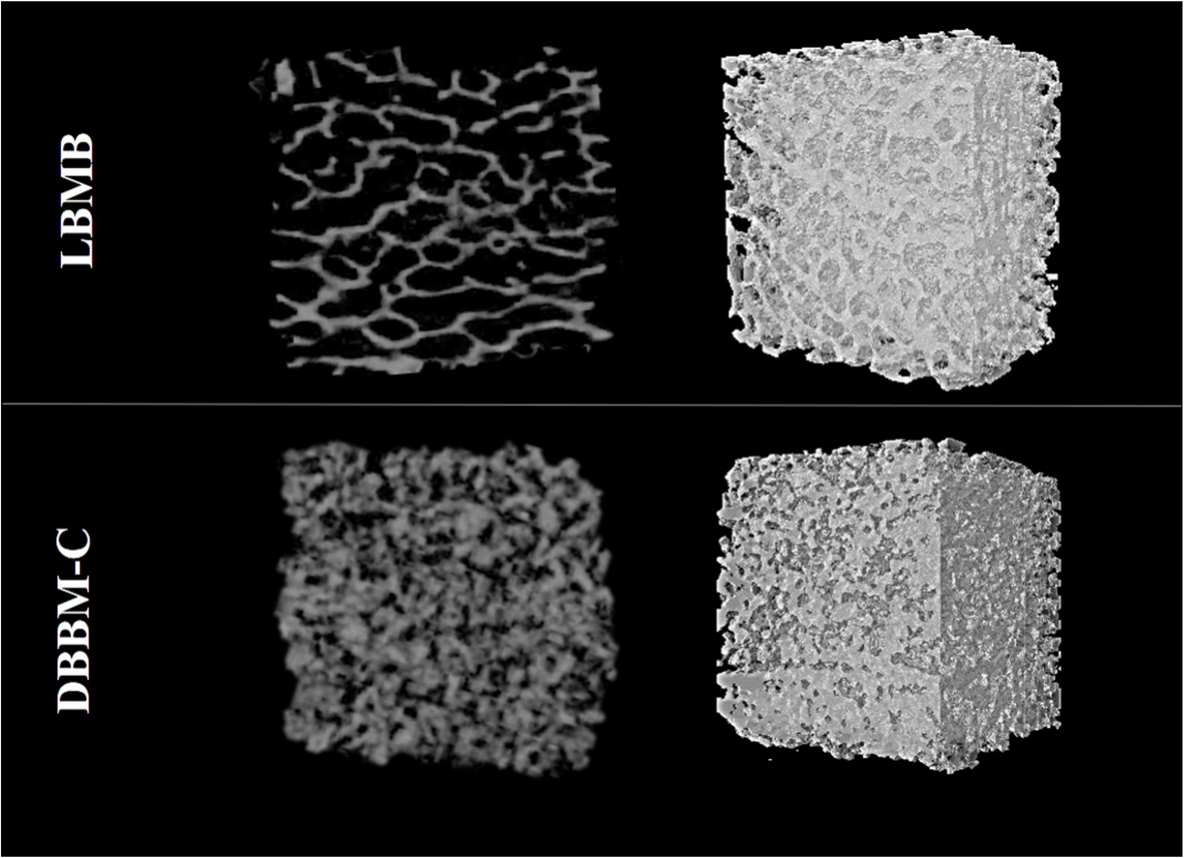
Micro-CT imaging of deproteinized bovine bone mineral with Collagen (DBBM-C), lyophilized bovine medullary bone (LBMB) blocks

The original DBBM-C graft is characterized by hydroxyapatite particles connected by collagen, marked by larger bone volume (↑42% BV/TV) and larger surface (↑28% BS/TV) compared to LBMB grafts (Fig. 2 and Table 1). However, no significant difference for trabecular thickness (Tb.Th) was found between both bone grafts.

**Table 1:** Descriptive analysis of morphometric parameters for the untreated bone defects and deproteinized bovine bone mineral with collagen (DBBM-C) and lyophilized bovine medullary bone (LBMB) for different post-operative periods

Morphometric parameters		Baseline line (T0)		3 months (T1)			6 months (T2)		
	Unit	DBBM-C	LBMB	Untreated	DBBM-C	LBMB	Untreated	DBBM-C	LBMB
Bone mineral density	mg HA/cm^3^	310.56 (44.52)^c^	286.24 (74.6)	442.55 (81.52)	362.88 (93.81)	368.62 (57.58)	419.82 (71.51)	442.1 (63.66)^c^	363.97 (5.05)
Bone volume fraction	(%)	53.86 (7.57)^a^	31.2 (6.68)^a^	22.47 (7.15)^b^	57.18 (16.57)^b,a^	38.94 (7.87)^b,a^	22.54 (6.52)	47.20 (25.64)	33.09 (12.46)
Bone surface density	(mm^2^/mm^3^)	6.55 (0.66)^a,c^	4.69 (1.02)^a,c^	1.72 (0.50)^b^	5.20 (0.85)^b^	4.25 (0.80)^b^	1.64 (0.29)^b^	3.76 (1.34)^b**,**c^	3.24 (0.64)^b**,**c^
Trabecular thickness	(mm)	0.27 (0.02)	0.22 (0.03)	0.47 (0.06)	0.37 (0.10)	0.37 (0.12)	0.58 (0.15)	0.44 (0.15)	0.38 (0.09)
Trabecular number	(1/mm)	1.19 (0.24)^a,c^	1.41 (0.26)^a,c^	0.47 (0.13)^b^	1.52 (0.19)^b**,**a^	1.10 (0.31)^b,a^	0.38 (0.04)^b^	1.04 (0.49)^b,c^	0.84 (0.19)^c^
Trabecular separation	(mm)	0.30 (0.10)^a^	0.51 (0.12)^a^	1.55 (0.47)^b^	0.35 (0.11)^b**,**a^	0.54 (0.15)^b,a^	1.31 (0.33)^b^	0.68 (0.48)^b^	0.80 (0.26)
Trabecular pattern factor	(1/mm)	− 5.76 (2.56)^a^	0.81 (1.14)^a^	0.42 (3.63)^b^	− 5.61 (4.86)^b^	0.4 (1.73)	2.61 (3.05)	− 3.52 (6.28)	1 (2.64)
Structural model index		− 0.38 (0.75)^a^	0.95 (0.27)^a^	0.57 (1.95)	− 1.39 (3.74)	1.09 (0.56)	1.80 (1.33)	− 0.54 (2.70)	1.25 (0.83)
Total porosity	(%)	46.13 (7.57)^a^	68.79 (6.68)^a^	77.52 (7.15)^b^	42.81 (16.57)^b,a^	61.05 (7.87)^a^	77.45 (6.52)	52.79 (25.64)	66.9 (12.46)
Connective density	(1/mm^3^)	20.62 (4.52)^a,c^	9.65 (6.58)^a^	1.63 (0.88)^b^	13.04 (6.14)^b^	6.94 (3.08)	1.24 (0.60)^b^	6.78 (4.32)^b,c^	3.87 (1.26)

^a^Post-hoc showed significant statistical difference at the same time point between the grafts (LBMB and DBBM-C)

^b^Post-hoc showed significant statistical difference at the same time point between the untreated group and the grafts (LBMB or DBBM-C)

^c^Post-hoc showed significant statistical difference within the same graft experimental group (LBMB or DBBM-C) in different follow up times

**Fig. 3:**
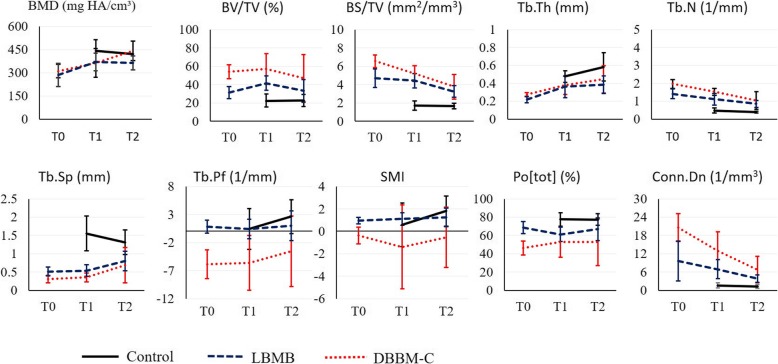
Morphometric analysis of the deproteinized bovine bone mineral with 10% collagen (DBBM-C) and deproteinized bovine medullary bone (LBMB) graft blocks at baseline (T0), in the region of the critical defects after 3 months (T1) and after 6 months (T2) post-operative follow-up. In the control group, the bone defects were left untreated. Morphometric parameters were subdivided in density-related parameters: bone mineral density (BMD in mg HA/cm^3^); quantity-related: bone volume fraction (BV/TV in %), bone surface density (BS/TV in mm^−1^), trabecular thickness (Tb.Th in mm); and structural-related parameters: trabecular number (Tb.N in 1/mm), trabecular separation (Tb. Sp in mm), trabecular pattern factor (Tb.Pf in 1/mm), structural model index (SMI), connective density (Conn.Dn in 1/mm^3^), total porosity percentage (Po[tot] in %)

In contrast, LBMB preserves the original trabecular bone structure, which explains its lower number of trabeculae (↓18% Tb.N), smaller number of connections (↑114% Tb.Pf and ↓53% Conn.Dn), and larger separation between them (↑49% Po[tot] and ↑70% Tb.Sp) compared to DBBM-C (Figs. 2 and 3 and Table 1). This is in line with the structure model index parameter that indicates a concave-like structure (SMI < 0) for DBBM-C and a plate-like structure (SMI = 1) for LBMB. No significant difference in  bone mineral density (BMD) was observed between the two grafts.

### Morphometric changes in the grafted defects

There was a significant main effect of graft type for bone replacement (*F* = 4.69; *p* < 0.01; *n*_p_^2^ = 0.37) and structural remodeling (*F* = 6.66; *p* < 0.001; *n*_p_^2^ = 0.65) parameters (Fig. 3). LBMB grafted regions were characterized by significantly less bone volume (↓51% BV/TV) and a smaller bone surface (↓25% BS/TV) over time compared to DBBM-C. No significant difference was observed for trabecular thickness. In general, LBMB showed smaller trabecular number (↓34% Tb.N) and connectivity (↓95% Conn.Dn and ↑797% Tb.Pf), resulting in higher porosity (↑37% Po[Tot]) in comparison to DBBM-C. In correspondence with the graft blocks in the baseline, the concave-like structure (SMI < 0) and the plate-like structure (SMI = 1) remained for DBBM-C and LBMB, respectively. No significant difference in BMD (*F* = 1.83; *p =* 0.19; *n*_p_^2^ = 0.07) was observed between LBMB and DBBM-C grafted regions over the postoperative periods.

There was as significant effect of postoperative follow-up time for bone replacement (*F* = 3.85; *p* < 0.003; *n*_p_^2^ = 0.32), structural remodeling (*F* = 2.62; *p* = 0.01; *n*_p_^2^ = 0.42), and BMD (*F* = 6.03; *p* = 0.007; *n*_p_^2^ = 0.31) parameters considering both grafts (Fig. 3 and Table 1). In comparison with baseline, after respectively 3 and 6 months, there was a significant remodeling indicated by an increased bone mineral density (↑23% and (↑35%), trabecular thickness (↑50% and (↑67%), and separation (↑10% and ↑83%). This is in line with the significant decrease in trabecular number (↓22% and ↓44%), connective density (↓34% and ↓65%), and bone surface density (↓16% and ↓38%).

The interaction between graft type and time was not significant (*p* > 0.05) for neither bone replacement, structural remodeling, and BMD parameters.

### Morphometric differences between bone grafts and untreated group

There was a significant main effect of group for morphometric bone replacement (*F* = 6.46; *p* < 0.001; *n*_p_^2^ = 0.38) and remodeling (*F* = 4.92; *p* < 0.001; *n*_p_^2^ = 0.51) parameters (Fig. 3). In comparison to the untreated group, DBBM-C and LBMB grafted defects were respectively characterized by higher bone volume (↑131% and ↑60% BV/TV) and more complex structure, presenting higher bone surface (↑166% and ↑122% BS/TV), thinner (↓28% and ↓21% Tb.Th), and more numerous trabeculae (↑194% and ↑124%), with higher number of connections (↓421% and ↓50% Tb.Pf; ↑580% and ↑271% Conn.Dn). In line with these results, both DBBM-C and LBMB grafted regions also presented smaller trabecular separation (↓63% and ↓53% Tb.Sp) and porosity (↓38% and ↓17% Po[Tot]) in relation to the controls.

In the control group, no significant effect of postoperative follow-up time was observed for bone replacement, remodeling and bone mineral density (*p* > 0.05).

### Interface graft-host

**Fig. 4:**
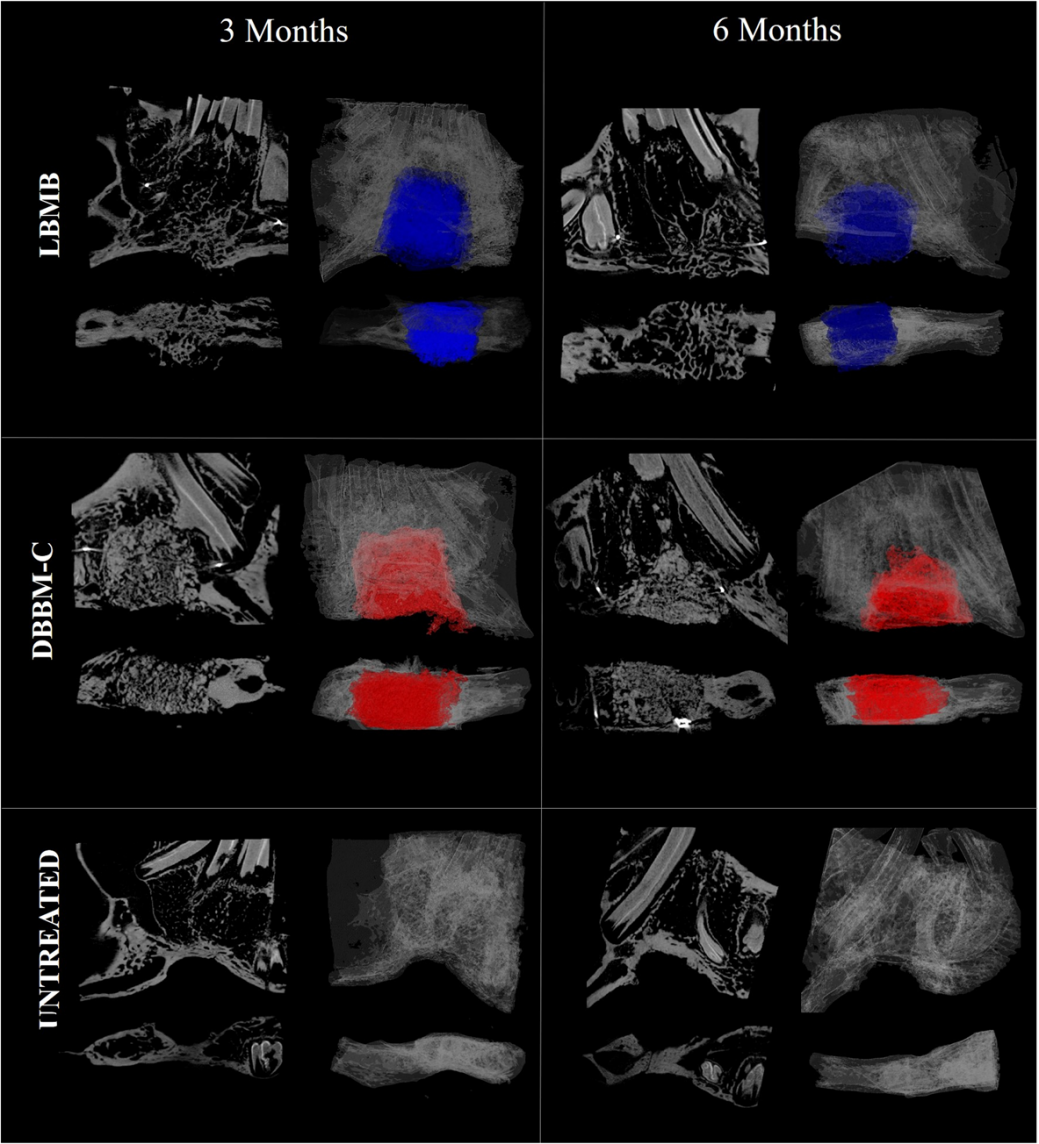
Micro-CT imaging of the bony defects filled with deproteinized bovine bone mineral with collagen (DBBM-C), lyophilized bovine medullary bone (LBMB)or left untreated after 3 or 6 months post-operative follow-up

All untreated defects were not completely filled with hard tissue after 6 months and showed a concave surface in the mandibular basal cortex (Fig. 4). Visually, it was possible to notice that the tissue repair process produced variable characteristics of the defect regions of each animal within the same graft group. While DBBM-C grafts adapted to the shape of the defect during repair, LBMB blocks preserved their initial square structure (Fig. 4).

In the DBBM-C group, a more corticalized bone structure was observed at the host–graft interface, while in the center of the grafted region the initial porous structure was maintained (Fig. 4). The original trabecular bone structure of LBMB grafts showed a clear integration with the adjacent host cortical bone, making it difficult to visually distinguish graft and host bone.

## Discussion

The aim of this study was to compare the morphometric characteristics and bone mineral density of DBBM-C and LBMB graft blocks. Additionally, bone replacement, remodeling, and bone mineral density of segmental defects filled with both xenografts were compared with untreated defects following 3- or 6-months postoperative period.

Original DBBM-C and LBMB blocks had a distinct morphometric structure due to the difference in processing. DBBM-C were characterized by hydroxyapatite particles bounded by collagen, while the LBMB blocks preserved the natural trabecular structure [5, 15]. Initial DBBM-C blocks presented denser (↑BV/TV), more complex (↑BS/TV, ↑Tb.N) and connected structure (↓Tb.Pf and ↑Conn.Dn), with lower porosity (↓Po[Tot]) and smaller pore size (↓Tb.Sp) compared to LBMB.

When comparing both blocks over time, a significant impact was induced by the grafts and the follow up time in the morphometric parameters. However, current results considering the interaction between graft and follow-up time showed no significant impact in bone replacement and remodeling morphometric parameters, suggesting similar changes for the DBBM-C and LBMB grafted regions. In relation to the untreated regions, both DBBM-C and LBMB grafts resulted in an increased bone replacement and structural complexity.

In clinical practice, guided bone regeneration with bone grafts frequently precedes implant placement in cases where insufficient bone support is available for functional rehabilitation [18, 26]. Bone microarchitecture is an important component for bone quality and may influence implant stability and rehabilitation success [24]. In accordance with our results, the microarchitecture of DBBM augmented bone has been documented as consisting of residual bone graft particles in close contact with newly formed bone leading to a dense structure [27]. These residual particles may remain unresorbed for long periods, leading to high stability in the maintaining of bone volume [27, 28]. It has been demonstrated that implant stability increases with denser bone (↑BV/TV, ↑Tb.Th, ↑Tb.N, and ↓BS/TV) plate-like structure (↓SMI) and smaller marrow spaces (↓Tb.Sp) [24]. Indeed, high implant success rates have been reported in DBBM grafted sites [29, 30].

In this experiment, DBBM-C blocks presented higher connective density (↑Conn.Dn and ↓Tb.Pf) compared to LBMB. This finding might have been influenced by the micro-CT segmentation. DBBM-C blocks are formed by bovine bone particles banded to each other by porcine collagen [8, 9]. The high number of particles close to each other may lead to a miss interpretation during segmentation. In this way, connections do not reflect a real trabecular connection node. The absence of real connections explains the changes in the DBBM block shape and the significant decrease in connectivity during healing. Differently, LBMB blocks connected trabecular structure may be responsible for maintenance of connectivity and block form during bone repair.

LBMB and DBBM-C grafts presented themselves with different host-bone interfaces. In LBMB group, graft and host bone seemed to be well integrated, making it impossible to define the exact borders. In contrast, in the DBBM-C group, a hypodense line between the graft and basal cortical was often present. The formation of a bond at the graft-host interface occurs as a result of a remodeling process, and is influenced by graft osteogenic, osteoinductive properties and resorption potential, [21, 31]. The resorption rates of bovine xenografts may vary greatly among commercially available materials, influenced both by graft structure and physico-chemical properties [5, 32].

The interconnection between the graft and the host is also influenced by graft porosity. The graft structure may present sufficient porosity, pore size, and interconnectivity to allow osteoconduction [21, 33]. An open porosity above 50% and pore sizes in the range of 200 to 800 μm are pointed out to be optimal for bone tissue ingrowth [21]. Scanning electron microscopy microstructural characterization of LBMB has shown 87–963 μm pore size while DBBM presented 20–200 μm pore size [15, 34]. Current results have shown that LBMB presents higher mean porosity and trabecular separation in comparison to DBBM-C and this difference is maintained in the grafted regions.

It is possible that the selection of the VOI may have influenced the morphometric quantification. This investigation was focused on the cross-sectional evaluation of the grated regions in different post-operatory moments, hence no initial imaging of the region of the defect was available for comparison and, in some cases, the exact edge of the defect was difficult to identify. To avoid mistakes, the same volume of interest considering the region of the defect was selected for all samples. The possible differences between bone architecture in the center and in the borders of the defect were not considered. Additionally, the formation of concave cortical bone in the region of the defect and within the VOI analyzed lead to an increased BMD in the untreated group in comparison to the grafted groups. Wong et al. (2010) demonstrated that the formation of new bone occurs in the DBBM-C grafted region periphery and tends to grow across the defect [10]. A similar bone neoformation pattern has also been demonstrated for LBMB [19]. In DBBM-C group, denser regions were observed in the periphery of the graft both in T1 and T2.

The results need to be interpreted in light of some limitations. Micro-CT is a non-destructive method that provides accurate 3D imaging of bone microstructure [35]. However, bone healing is a dynamic process that is influenced by several different graft material properties that should also be considered [36]. Histological investigation of the morphometric parameters will be the focus of further investigation. Future studies may also consider an in vivo imaging follow up of the grafted regions to allow the identification of early changes.

In this investigation, a segmental defect was constructed in the rabbit’s jaw [37]. Animal models play an indispensable role to understand bone graft materials properties, such as osteoconductivity, biocompatibility, resorption, and interaction with host tissues [31]. Metabolic rate of the test animal, characteristics of the created defect, and anatomical location should be considered when evaluating the results [38]. No significant changes in bone quantity, density, and structure were observed in the non-treated defects in the interval between 3 and 6 months suggesting that segmental bone defect healing in this rabbit model have been close to completion already at 3 months.

## Conclusion

Although DBBM-C and LBMB blocks had a distinct morphometric structure, LBMB grafts showed a similar bone replacement and structural remodeling as DBBM-C. Both graft materials showed an enhanced bone formation and more complex structure compared to untreated defects. Therefore, both graft are indicated for guided bone regeneration procedures in the maxillofacial region.

## Data Availability

The datasets used and/or analysed during the current study are available from the corresponding author on reasonable request.
